# Numerical Modeling Calcium and CaMKII Effects in the SA Node

**DOI:** 10.3389/fphar.2014.00058

**Published:** 2014-04-01

**Authors:** Yael Yaniv, Victor A. Maltsev

**Affiliations:** ^1^Laboratory of Cardiovascular Science, Intramural Research Program, National Institute on Aging – National Institutes of HealthBaltimore, MD, USA; ^2^Department of Biomedical Engineering, Technion – Israel Institute of TechnologyHaifa, Israel

**Keywords:** cardiac pacemaker, sinoatrial node, numerical modeling, calcium, CaMKII, ion channels

## Abstract

Sinoatrial node (SAN) is the primary heart pacemaker which initiates each heartbeat under normal conditions. Numerous experimental data have demonstrated that Ca^2+-^ and CaMKII-dependent processes are crucially important for regulation of SAN cells. However, specific mechanisms of this regulation and their relative contribution to pacemaker function remain mainly unknown. Our review summarizes available data and existing numerical modeling approaches to understand Ca^2+^ and CaMKII effects on the SAN. Data interpretation and future directions to address the problem are given within the coupled-clock theory, i.e., a modern view on the cardiac pacemaker cell function generated by a system of sarcolemmal and intracellular proteins.

## INTRODUCTION

**FIGURE 1:**
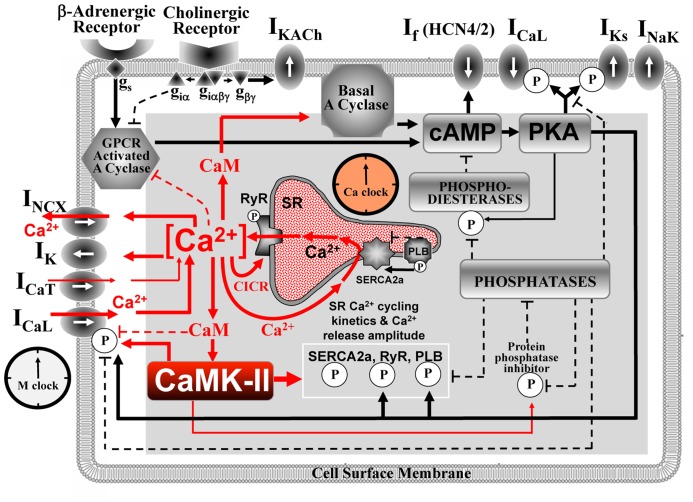
**Schematic illustration of sinoatrial node cell as a coupled-clock system of voltage membrane clock (M-clock) and an intracellular, sarcoplasmic reticulum (SR)-based Ca^**2**+^-clock (gray intracellular area**). Interactions of key molecules comprising the system with Ca^2+^ and CaMKII signaling are shown in red. Note that common regulatory factors govern the function of both clocks. These common factors, including CaMKII, act as nodes within the system to couple the function of both clocks activities. The system is balanced: signals accelerating action potential (AP) firing are balanced by signals suppressing AP firing. This balance determines a given steady-state level of net Ca^2+^, cAMP, and protein phosphorylation via PKA, and CaMKII. G protein-coupled receptors (top left corner) within the cell membrane modulate both the Ca^2+^-clock and M-clock function via the same crucial signaling nodes of the system. Modified from Lakatta et al. (2010).

Under normal conditions, SAN cells (SANC) generate spontaneous rhythmic action potentials (AP) that initiate the heartbeat. The evolution of thought regarding the cardiac pacemaker cell operation paradigm switched back and forth between intracellular origin [e.g., a “metabolic” intracellular clock (Bozler, 1943) or sarcoplasmic reticulum (SR)-based Ca^2+^-clock (Maltsev et al., 2006)] and cell membrane origin [voltage membrane clock or M-clock (Noble, 1960)]. A more recent paradigm shift has been the realization that both intracellular and sarcolemmal mechanisms are tightly, dynamically coupled to each other and are indispensable for normal pacemaker function. These ideas have been summarized within a “coupled-clock” theory of interacting M-clock and Ca^2+^-clock (Maltsev and Lakatta, 2009; **Figure 1**) that explained numerous experimental findings (Lakatta et al., 2010; Maltsev and Lakatta, 2012). The key processes of the coupled-clock system depend on Ca^2+^, calmodulin (CaM), and CaMKII signaling (**Figure 1**, red). Interactions within the system are extremely complex and their detailed investigation requires numerical model simulations. The CaMKII function in pacemaker cells has not been systematically studied using numerical simulations. Our review summarizes major principles of the coupled-clock theory, available data, and existing numerical modeling approaches that are important to delineate future numerical integration and exploration of CaMKII within the pacemaker cell system.

## INTEGRATION OF Ca^2+^ AND CAMKII SIGNALING WITHIN THE COUPLED-CLOCK SYSTEM

Operation of the coupled-clock system has been explored in recent numerical model studies (Maltsev and Lakatta, 2009, 2013; Yaniv et al., 2013a, d), and experimental evidence for the coupled-clock theory has been summarized (Lakatta et al., 2010; Maltsev and Lakatta, 2012). The system generates spontaneous, rhythmic APs separated by a slow diastolic depolarization (DD) that starts each cycle from the maximum diastolic potential (MDP ~-60 mV) and brings the membrane potential (V_ m_) to a cell excitation threshold of ~-40 mV. The coupled-clock theory postulates that the DD is generated by the two coupled oscillators, Ca^2+^-clock and M-clock, rather than just by M-clock alone (**Figure 1**).

**FIGURE 2:**
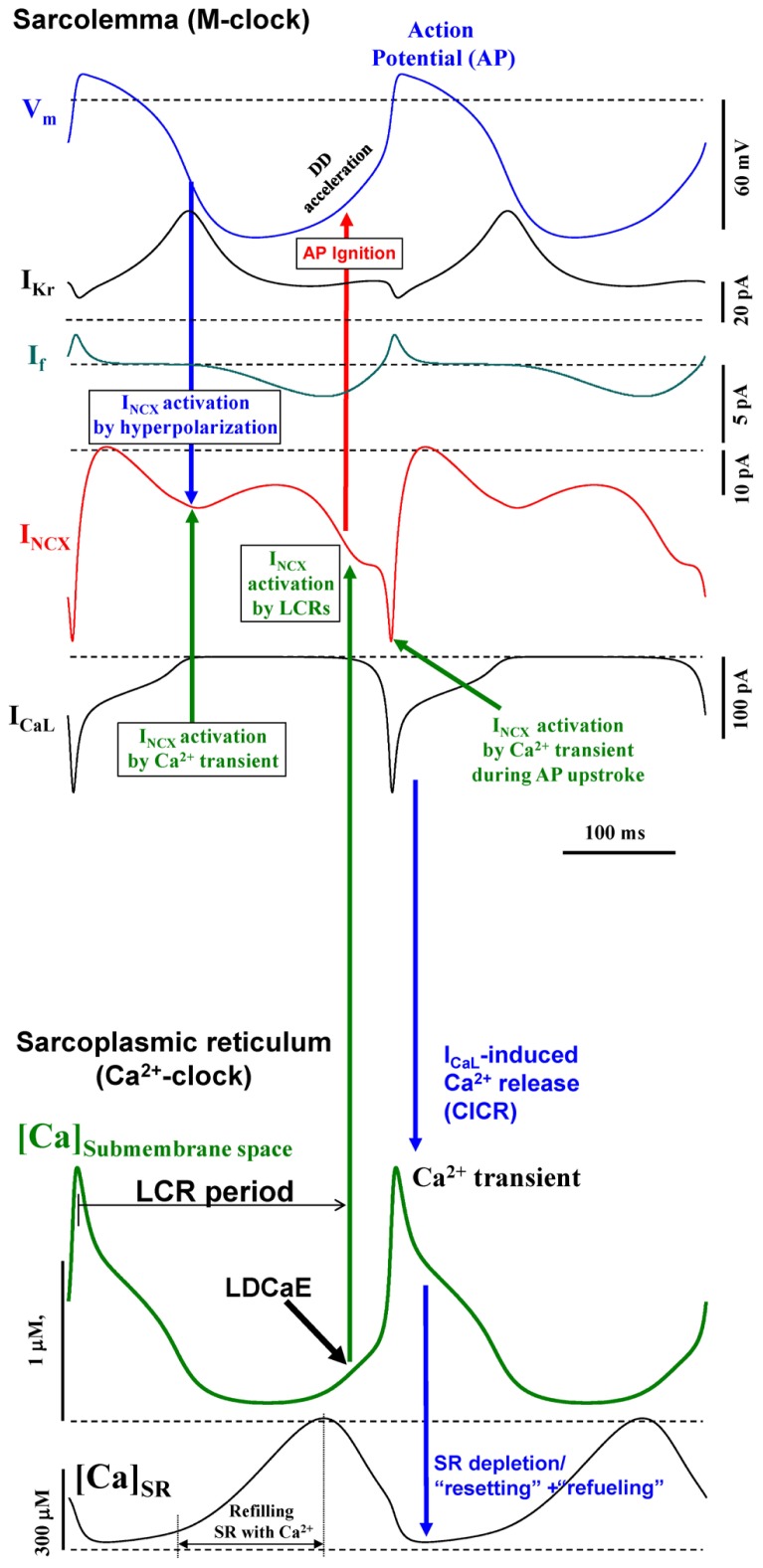
**Coupled-clock Maltsev–Lakatta numerical model (Maltsev and Lakatta, 2009) predicts complex synergistic interactions between cell membrane and Ca^**2**+^ cycling proteins within SANC (see “Integration of Ca^**2**+^ and CaMKII Signaling within the Coupled-Clock System”)**. Modified from Maltsev and Lakatta (2009).

The first numerical model of M-clock was developed by Noble (1960), by application of Hodgkin–Huxley (HH) theory to cardiac pacemaker cells. The M-clock-based models generate the DD via time-dependent kinetics of ion channels upon AP repolarization, e.g., by inactivation of a K^+^ current (Noble, 1960) or by activation of a non-selective, “funny” current (DiFrancesco and Noble, 2012). The SR, a major Ca^2+^ store in cardiac cells, can also generate spontaneous oscillations via rhythmic cycles of SR Ca^2+^ pumping (via SERCA) and release (via release channels, RyR; **Figure 1**). Ventricular muscle cells can spontaneously cycle Ca^2+^ (under conditions of high Ca^2+^ loading) via global Ca^2+^ waves via regenerative Ca^2+^-induced Ca^2+^ release (CICR) propagating by Ca^2+^ diffusion (Fabiato, 1983). However, cardiac pacemaker cells generate rhythmic, spontaneous Ca^2+^ releases during DD under normal Ca^2+^ conditions (in the absence of Ca^2+^ overload; Huser et al., 2000; Bogdanov et al., 2001). These releases occur in the form of abrogated waves, dubbed local Ca^2+^ releases or LCRs. The synchronous occurrence of the LCRs generates a powerful, diastolic, net Ca^2+^ signal, dubbed the late diastolic Ca^2+^ elevation or LDCaE (**Figure 2**). The rhythmic LCRs are generated in the absence of M-clock, e.g., under voltage clamp or in membrane-permeabilized SANC [when [Ca^2+^] is normal, ~100 nM, review (Lakatta et al., 2010)]. The Ca^2+^-clock in SANC is driven by Ca^2+^ cycling proteins (e.g., phospholamban and RyR, **Figure 1**), whose function is enhanced by phosphorylation via basal activity of PKA (Vinogradova et al., 2006) and CaMKII (Vinogradova et al., 2000). In turn, the PKA is activated by a high basal level of cAMP produced by Ca^2+^-activated adenylyl cyclases (ACs) which are highly expressed in SANC [particularly types 1 and 8, (Mattick et al., 2007; Younes et al., 2008)]. The high rate of cAMP production and protein phosphorylation is counterbalanced by activities of phosphatases and phosphodiesterases. Interestingly, a powerful Ca^2+^-clock generating rhythmic LCRs (similar to that in SANC) also emerges in ventricular myocytes when the phosphorylation of Ca^2+^ cycling protein increases (e.g., via inhibition of phosphatases and/or phosphodiesterases; Sirenko et al., 2014).

The Ca^2+^-clock and the M-clock are coupled in SANC via Na^+^/Ca^2+^ exchanger (NCX; **Figure 2**) that senses the LCR ensemble (i.e., LDCaE) and, operating in the forward mode, generates a substantial inward current (I_ NCX_) during DD. M-clock, in turn, regulates Ca^2+^-clock via L-type Ca^2+^ current (I_ CaL_) by (1) resetting phases of local Ca^2+^ oscillators that synchronizes LCR ensemble; (2) supplying Ca^2+^, i.e., the Ca^2+^-clock’s oscillatory substrate. Both clocks are coupled not only directly via V_ m_ and Ca^2+^, but indirectly, enzymatically, by coupling factors, such as PKA and CaMKII, affecting multiple targets within both clocks (**Figure 1**). PKA- and CaMKII-dependent phosphorylation enhances function of the proteins comprising the system and is required for normal pacemaker function and autonomic modulation. Because of these complex interactions (which define the Ca^2+^ balance and enzymatic activity balance), each component of the system contributes to the LCR spatiotemporal characteristics, especially the LCR period, i.e., the time when LCRs emerge and accelerate the DD (**Figure 2**). Thus, the LCR period is contributed not only directly by the Ca^2+^ release channels RyR, but also indirectly by L-type Ca^2+^ channels (LCCh), SERCA, and NCX regulating Ca^2+^ fluxes (Maltsev et al., 2013), and even by K^+^ channels or “funny” channels via respective V_ m_ changes, also regulating Ca^2+^ fluxes (Yaniv et al., 2013a).

## EXPERIMENTAL EVIDENCE FOR IMPORTANCE OF CaMKII SIGNALING IN PACEMAKER CELLS

CaMKII indirectly senses [Ca^2+^] by binding Ca^2+^-CaM complex at the CaM region in its regulatory domain, which increases its activity (Anderson et al., 2011). (Of note, there are two predominant CaMKII isoforms in the heart: CaMKIIδ_ B_ localizes in nuclei and CaMKIIδ_ C_ in cytosol). While CaMKII does not regulate directly cAMP production, reduction in CaMKII activity is associated with reduction in [cAMP] in rabbit SANC (Yaniv et al., 2013b), indicating a complex interplay of the CaMKII, ACs, and PKA signaling (**Figure 1**). In this special issue Wu and Anderson discuss in detail experimental evidence for contribution of CaMKII activity to SAN function during health and heart disease (Wu and Anderson, 2014). Here we summarize the key facts with respect to the integration of CaMKII within the coupled-clock system of SANC (**Figure 1**) and its future numerical modeling.

Pharmacological inhibition of CaMKII signaling (using AIP or KN-93) depresses the basal rate and amplitude of spontaneous APs in SANC of rabbit (Vinogradova et al., 2000; Yaniv et al., 2013b) and guinea-pig (Rigg et al., 2003). Confocal imaging of immunolabeled proteins demonstrates that active CaMKII is highly localized beneath the surface membrane (Vinogradova et al., 2000). Thus, CaMKII activity is geographically associated with proteins of both M- and Ca^2+^-clocks. CaMKII modulates several membrane ion channels in the heart: LCCh, K^+^ channels, and Ca^2+^-clock proteins: SERCA (directly and indirectly via phospholamban) and RyR. Studies in isolated rabbit SANC suggested that CaMKII regulates the pacemaker activity via modulating I_ CaL_ inactivation and reactivation (Vinogradova et al., 2000) and LCR morphology (Vinogradova et al., 2011). *I*_ f_ is not affected directly by CaMKII inhibition (Rigg et al., 2003).

Thus, contribution of CaMKII to basal AP generation by SANC was demonstrated for rabbit and guinea pig [but remains controversial for mice (Zhang et al., 2005; Wu et al., 2009)]. Because CaMKII is sensitive to the frequency of the Ca^2+^ transients, CaMKII is ideally suited to respond to changes in SAN rhythm. For example, electrical stimulation alone increases CaMKII-dependent phosphorylation of phospholamban at CaMKII phosphorylation site in a frequency-dependent manner in ventricular myocytes (Hagemann et al., 2000). CaMKII also mediates SAN response to β-adrenergic receptor stimulation (Wu et al., 2009). Moreover, SANC and isolated hearts from mice with CaMKII inhibition (by transgenic expression of AC3-I) were insensitive to BayK, an LCCh agonist, which increases pacemaker rate in wild type mice (Gao et al., 2011). New evidence that CaMKII is a key part of the coupled-clocked system (**Figure 1**) has been obtained in studies of specific *I*_ f_ inhibitor ivabradine (Yaniv et al., 2013a; discussed below).

CaMKII activity can also be enhanced by pro-oxidant conditions (Erickson et al., 2008). Clinical studies show that right atrial tissue from patients with heart failure who also required artificial pacemakers have more Oxidize-CaMKII compared to patients with heart failure alone and patients without heart failure or severe SAN dysfunction (Swaminathan et al., 2011). Ang II infusion in mice increases Oxidize-CaMKII and elicits SAN dysfunction that is prevented by overexpression of a synthetic CaMKII inhibitory peptide (AC3-I) or by CaMKIIN, an endogenous CaMKII protein present in neurons, but absent in the heart (Swaminathan et al., 2011).

CaMKII activity appears to be increased in heart disease (e.g., arrhythmia, heart failure, atrial fibrillation; Anderson et al., 2011). Sinus sick syndrome prevails during heart failure and hypertension conditions (with both conditions exhibiting elevated angiotensin II levels). Because CaMKII inhibition is sufficient to protect against angiotensin II-induced sick sinus syndrome in aforementioned mouse model (Swaminathan et al., 2012), CaMKII inhibition may be a useful approach to prevent sinus sick syndrome.

It was demonstrated that basal AC-cAMP/PKA signaling directly, and Ca^2+^ indirectly, regulate mitochondrial ATP production (Yaniv et al., 2011, 2013c). As a crucial element of normal automaticity in rabbit SANC, CaMKII signaling is also involved in SANC bioenergetics. When ATP demand is reduced by interfering with CaMKII or CaM activity, SANC become depleted of ATP, indicating reduction in ATP generation with lower demand (Yaniv et al., 2013b).

## NUMERICAL MODELING STUDIES THAT SHOW IMPORTANCE OF CaMKII SIGNALING FOR SAN FUNCTION

Although CaMKII signaling, *per se*, has not been systematically studied in pacemaker cell models, at least two recent numerical model studies point to a key functional importance of CaMKII signaling in pacemaker cells and tissues.

Yaniv et al. (2013a) have recently demonstrated that CaMKII likely serves as a key functional integrator of M-clock and Ca^2+^-clock signals (**Figure 1**) by testing effects of specific perturbations of either clock in rabbit SANC. The M-clock was specifically perturbed by ivabradine that at low concentrations (<3 μM) specifically inhibits I_ f_, i.e., it does not suppress I_ CaL_ (Yaniv et al., 2012a), other membrane ion currents (Bois et al., 1996), or Ca^2+^ cycling in permeabilized SANC (Yaniv et al., 2013a).

Numerical simulations (Yaniv et al., 2013a) using a modified coupled-clock Maltsev–Lakatta model (Yaniv et al., 2012b), provided new insights in ivabradine-induced bradycardia. An initial *I*_ f_ reduction slows AP rate that, in turn, reduces the number of I_ CaL_ activations/unit time, average Ca^2+^ influx, and Ca^2+^ available for SR pumping. This results in lower SR Ca^2+^ load and longer LCR period (both effects were also found experimentally). Later activation of diastolic I_ NCX_ by the LCRs (and I_ NCX_-linked DD acceleration) leads to a delayed activation of I_ CaL_, i.e., M-clock slowing. Thus, inhibition of the M-clock inhibits (indirectly) Ca^2+^-clock that further suppresses the M-clock, and so on, until the coupled-clock system attains a new steady-state.

Interestingly, model simulations show that the complex ivabradine effects extend further, beyond “biophysical” entrainment, and likely include an additional “biochemical” component. The aforementioned decrease in average Ca^2+^ influx produced by ivabradine not only decreases Ca^2+^ available for SR pumping, but also likely decreases protein phosphorylation signaling via Ca^2+^-activated-CaMKII and Ca^2+^-activated-ACs-cAMP/PKA pathways. This leads to further reductions in the average Ca^2+^ influx and, therefore, SR Ca^2+^ loading and AP firing rate. Simultaneously, reduction in cAMP shifts the *I*_ f_ activation curve (effecting further M-clock slowing). If the “biochemical” crosstalk is lacking, model simulations (Yaniv et al., 2013a) predict only about 50% of the experimentally measured bradycardia produced by ivabradine. Thus, the entire ivabradine effect is explained by a crosstalk of equally important biophysical and biochemical mechanisms (including CaMKII signaling).

According to the coupled-clock theory (Maltsev and Lakatta, 2009) any selective perturbation of either clock will inevitably affect the function of the other and the entire coupled-clock system. In line with this postulate, the bradycardic effect is symmetric: it does not depend on which clock was initially perturbed. Both the LCR period and AP cycle length become prolonged by either perturbations of M-clock (e.g., using ivabradine) or Ca^2+^-clock (e.g., using cyclopiazonic acid to selectively inhibit SERCA), with the LCR period reporting the resultant complex effect (Yaniv et al., 2013a).

Heart rate reductions produced by ivabradine or HCN4 mutations have been interpreted as a pure result of insufficient *I*_ f_ function. However, based on the results discussed above, these effects are likely complex, involving the secondary changes in Ca^2+^-clock and the entire coupled-clock system (that includes CaMKII signaling; Yaniv and Lakatta, 2013). Effects of mutations of Ca^2+^ cycling proteins on pacemaker function also likely include clocks coupling, i.e., secondary effect on *I*_ f_ (via Ca^2+^-activated-ACs and cAMP), rate-dependent effects on both clocks, ultimately resulting in mutual entrainment of the clocks (Yaniv and Lakatta, 2013; Yaniv et al., 2013a).

Luo et al. (2013) numerically modeled a further level of CaMKII effects related to cell death that is important to approach the mechanisms of insufficient pacemaker function in disease and aging. They developed a two-dimensional histologically reconstructed mathematical model that takes into account SAN cell death and fibrosis expressed in myocardial infarction by oxidizing CaMKII. Their simulations predict decreased conduction velocity and shift of the leading pacemaker site under these conditions. Thus, changes in CaMKII signaling can result in morphological changes of the SAN tissue which can affect cardiac impulse initiation.

## LOCAL Ca^2+^ AND CaMKII SIGNALING IN PACEMAKER CELLS

The local Ca^2+^ control theory (Stern, 1992) remains a key in understanding the mechanisms of cardiac excitation-contraction coupling. This theory explained graded CICR phenomenon via statistics of success and failure of an initiating event (such as LCCh opening) to recruit stochastic Ca^2+^ release units (CRUs) to fire. While partially periodic LCRs (comprising Ca^2+^ clocks) in cardiac pacemaker cells are generated by the CRUs, they are, in fact, a product of complex local interactions of proteins residing in both cell membrane and the SR, i.e., RyR, SERCA, LCCh, and NCX. These interactions, in turn, are regulated by PKA and CaMKII signaling (**Figure 1**).

During the last decade mathematical models have been developed in ventricular myocytes to describe the CaMKII effects via regulation of ionic currents (Hund and Rudy, 2004; Grandi et al., 2007). More recent models describe CaMKII activity as a function of subspace Ca^2+^, CaM, and phosphatase activity (Saucerman and Bers, 2008). These studies have demonstrated that the different affinities of CaM and CaMKII and calcineurin determine their sensitivity to local versus global Ca^2+^ signals that regulates excitation-contraction coupling. Hashambhoy et al. (2009) developed a stochastic model describing the dynamic interactions among CaMKII, LCCh, and phosphatases as a function of dyadic Ca^2+^ and CaM levels.

Local Ca^2+^ mechanisms have been recently modeled in pacemaker cells. The LCRs are generated via stochastic recruitment of the neighboring CRUs (Maltsev et al., 2011) regulated by local interactions of RyR, SERCA, and NCX (Maltsev et al., 2013). Some irregularity in RyR spatial distribution is not an imperfection, but rather a functional modality of the pacemaker cells [abstract (Maltsev et al., 2014)]. The irregularity decreases nearest neighbor-to-neighbor distances among the CRUs and thereby facilitates local CICR forming wavelet-like LCRs. This new local control mechanism regulates the balance between robustness and flexibility of pacemaker cell function.

The most advanced SANC model (Stern et al., 2014) features stochastic propagated spontaneous diastolic Ca^2+^ release in three dimensions. This model describes explicit gating of individual Ca^2+^ channels (both RyR and LCCh), without assuming either a discrete sub-membrane compartment or an inactivated state of the RyR. The model succeeded in reproducing observed propagating local Ca^2+^ releases and realistic pacemaker rates only when RyR locations were assigned taking into account irregular, hierarchical distribution of RyR clusters (small and large) observed in 3D confocal scan sections of immunofluorescence staining. When the RyR sensitivity is very high or the NCX density is low, synchronization is lost, causing sympathetic stimulation to reduce (rather than increase) beating rate, often exhibiting arrhythmias (Maltsev et al., 2013; Stern et al., 2014). This regime may be important for rhythm abnormalities caused by heart failure, RyR mutations, or pharmacological NCX blockade.

Compared to previous models, lacking local Ca^2+^ dynamics (i.e., “common pool” models [Kurata et al., 2002; Maltsev and Lakatta, 2009)], the new models provide mechanistic insights into local crosstalk of the key molecules of the system: recruitment of RyRs (generating diastolic LCRs), RyR-LCCh and RyR-NCX crosstalk, and efficient SERCA operation (Maltsev et al., 2013). Indeed, Ca^2+^ signals within LCRs exhibit much higher amplitudes vs. those predicted by “common pool” models (~tens of μM vs. ~1 μM). Thus, the “local” models, predicting the realistic scale of Ca^2+^ signals within the inhomogeneous signaling network of SANC, seem to be a better choice to explore CaMKII effects in future studies of pacemaker cells.

## SUMMARY

In this review we have summarized the present state of experimental and numerical modeling studies on Ca^2+^ and CaMKII roles in cardiac pacemaker cells. Taking into account emerging importance of local Ca^2+^ control in cardiac pacemaker cells and also importance of local CaMKII signaling (reported in ventricular myocytes), accurate interpretation of experimental data on CaMKII effects in pacemaker cells will likely require integration of local (Saucerman and Bers, 2008) and molecular (Hashambhoy et al., 2009) mechanisms into new pacemaker cell models. Another important aspect that needs numerical integration is CaMKII involvement in SANC bioenergetics (Yaniv et al., 2013b). The new experimental studies combined with new model simulations will explore CaMKII interactions (**Figure 1**, red) with key regulatory molecules (e.g., ACs, PDEs, phosphatases, PKA, phospholamban), effector molecules (RyR, SERCA, NCX, LCCh, NCX, etc), and energy production of the system. This knowledge will contribute greatly to our understanding of cardiac impulse initiation and specific role of CaMKII signaling in the pacemaker regulation.

## AUTHOR CONTRIBUTIONS

Both authors contributed to the conception of the work, drafted the paper, approved the version to be published, and are accountable for all aspects of the work.

## Conflict of Interest Statement

The authors declare that the research was conducted in the absence of any commercial or financial relationships that could be construed as a potential conflict of interest.
